# Optimized Protocol for RNA Isolation from *Penicillium* spp. and *Aspergillus fumigatus* Strains

**DOI:** 10.3390/cimb46110778

**Published:** 2024-11-17

**Authors:** Aleksandra Siniecka-Kotula, Martyna Mroczyńska-Szeląg, Anna Brillowska-Dąbrowska, Lucyna Holec-Gąsior

**Affiliations:** Faculty of Chemistry, Department of Biotechnology and Microbiology, Gdansk University of Technology, 80-233 Gdańsk, Poland; aleksandra.siniecka-kotula@pg.edu.pl (A.S.-K.); martyna.mroczynska@pg.edu.pl (M.M.-S.); anna.brillowska-dabrowska@pg.edu.pl (A.B.-D.)

**Keywords:** RNA isolation, molds, RNA isolation efficiency

## Abstract

Efficient RNA isolation from filamentous fungi is crucial for gene expression studies, but it poses significant technical challenges due to the robust cell walls and susceptibility of RNA to degradation by ribonucleases. This study presents the effectiveness of two RNA isolation protocols for four species of filamentous fungi: *Penicillium crustosum*, *Penicillium rubens*, *Penicillium griseofulvum*, and *Aspergillus fumigatus*. Both protocols utilized Fenzol Plus for cell lysis but varied in the mechanical disruption methods: bead-beating versus manual vortexing. The results show that the bead-beater method (Protocol 1) yielded significantly higher RNA quantities, with better purity and integrity, as demonstrated by higher A260/A280 and A260/A230 ratios. RNA concentrations ranged from 30 to 96 µg/g of dry biomass in *Penicillium* species and up to 52 µg/g in *A. fumigatus*. The use of chloroform in Protocol 1 also enhanced RNA purity, effectively separating contaminants such as DNA, proteins, and polysaccharides. This optimized protocol is highly efficient and can be applied in routine laboratories handling large numbers of fungal samples, making it a robust method for downstream applications such as cDNA synthesis and transcriptome analysis.

## 1. Introduction

The isolation of nucleic acids, including RNA, is a critical step in molecular research on eukaryotic organisms such as fungi. RNA is a fundamental component in gene expression studies, as it enables transcriptome analysis and provides insights into gene regulation under various environmental conditions [1]. RNA isolation from microorganisms is a much more challenging and demanding process compared to DNA isolation. This is primarily due to the fact that RNA is highly susceptible to degradation by ubiquitously present ribonucleases. Additionally, RNA isolation from filamentous fungi introduces further complications in laboratory studies, stemming from their morphology and the structure of their cell walls. Due to the complex structure of fungal cells, which includes a robust cell wall composed of chitin and glucans, RNA isolation from these organisms poses a significant technical challenge [2].

Meanwhile, the quantity, quality, and integrity of the isolated RNA are crucial for its use in further studies, such as gene expression analysis. For the isolated RNA to serve as a template for creating a cDNA library, it must be free of DNA contamination, and the presence of proteins and salts in the isolate must be minimized [3,4]. Given that cDNA synthesis using commercially available kits requires at least 1 µg of RNA, achieving adequate RNA isolation efficiency is essential. Additionally, the integrity of the isolated RNA is crucial to prevent the formation of artifacts during subsequent analyses. Due to these challenges, a variety of RNA isolation techniques have been developed, each tailored to the specific needs and structural characteristics of individual fungal species [5].

In the scientific literature, three main approaches to RNA isolation from filamentous fungi are described: mechanical, chemical, and enzymatic methods for cell wall disruption. These methods are often used in combination to enhance the efficiency and quality of the RNA obtained [6]. One of the most common approaches involves the use of mechanical methods for disrupting the fungal cell wall, such as homogenization with ceramic or glass beads. This procedure is often performed in buffers containing guanidinium thiocyanate or other RNase inhibitors to prevent RNA degradation. Mechanical homogenization ensures the physical disruption of the robust cell wall, which is particularly effective for species with an exceptionally thick chitin layer. The average yield of RNA isolation using this method ranges from 50 to 100 µg of RNA per gram of dry fungal biomass, depending on the species and the homogenization parameters [5]. The advantage of this approach is its versatility; however, the risk of the mechanical degradation of nucleic acids may result in reduced RNA integrity, limiting its suitability for certain analyses.

Chemical methods of RNA isolation primarily rely on the use of strong denaturants such as phenol/chloroform, guanidinium thiocyanate, and detergents. The Chomczynski and Sacchi method (1987) remains one of the most commonly used chemical techniques, enabling the effective separation of RNA from DNA and proteins [6]. The efficiency of this method depends on the specific protocol used but generally yields between 30 and 70 µg of RNA per gram of dry fungal biomass. While this method is relatively inexpensive and straightforward, it requires great caution due to the use of toxic substances, which can impact laboratory safety [7].

RNA isolation can also be enzymatically assisted, utilizing enzymes that selectively degrade components of the cell wall, facilitating the release of genetic material. The yield of RNA obtained through this method is highly dependent on the specific fungal species; however, with the proper optimization of the process, yields ranging from 40 to 90 µg of RNA per gram of dry biomass can be achieved. Enzymatic digestion helps avoid the mechanical degradation of RNA but may require longer preparation times, and the enzymatic efficiency depends on the cell wall composition of the particular species [8]. Enzymes such as chitinases and glucanases are effective in gently treating cells; however, their efficiency is limited when dealing with highly diverse cell wall structures.

In practice, RNA isolation from filamentous fungi often requires a combination of the aforementioned methods. A combination of mechanical cell wall disruption with enzymatic assistance, along with the use of chemical denaturants, frequently becomes the optimal choice. This approach allows for the extraction of pure and intact RNA, suitable for further analyses such as RT-qPCR or RNA-Seq [9]. The yield of these complex protocols typically ranges from 60 to 120 µg of RNA per gram of dry fungal biomass, making them more efficient than individual approaches. An important step is also the elimination of DNA, which is usually achieved by the application of DNases to prevent contamination of the RNA by genomic DNA [7].

The choice of RNA isolation method depends on various factors, including the specific morphological characteristics of the fungi, cultivation conditions, and the target analysis. Contemporary research focuses on optimizing protocols to minimize time and costs while ensuring high-quality RNA yields. For example, Lange et al. (2018) demonstrated that the use of modern commercial kits, which combine both mechanical and chemical methods, can significantly reduce the preparation time and improve the RNA extraction efficiency from various fungal species [9]. The RNA yield from these kits can reach up to 150 µg per gram of dry biomass, representing a significant improvement compared to traditional methods.

In this article, the RNA isolation efficiency from fungi of the genus *Penicillium* sp. and *Aspergillus fumigatus* was compared using commercially available RNA isolation kits. Modifications were made to the protocols to account for the specific characteristics of fungal material, particularly the filamentous nature of the fungi.

## 2. Materials and Methods

### 2.1. Isolates and Culture

The study involved four species of filamentous fungi: three from the genus *Penicillium*—*P. crustosum*, *P. rubens*, and *P. griseofulvum*—as well as *Aspergillus fumigatus*. Two different strains of each species were analyzed. The strains studied were obtained from the resources of the Department of Biotechnology and Microbiology at Gdańsk University of Technology.

To prepare the mycelium for RNA isolation, a fragment of mycelium from the agar culture was used to inoculate 10 mL of Sabouraud broth (POL-AURA, Gdansk, Poland), with nine test tubes prepared for each species. The cultures were then incubated for 5 days at 27 °C. After the incubation period, the cultures were centrifuged for 10 min at 1800× *g*. The entire mycelium was then transferred to 2 mL test tubes with 1 g zirconium beads (diameter 1 mm) (A&A Biotechnology, Gdansk, Poland) using disposable loops. RNA was isolated according to the protocols outlined below.

### 2.2. Protocol No. 1

The RNA isolation was performed using the commercial Bead-Beat Total RNA Mini kit (A&A Biotechnology, Gdansk, Poland). For the disruption of the fungal mycelium, after adding 800 µL of Fenozol Plus (A&A Biotechnology, Gdansk, Poland), the samples were placed in a bead-beater device and shaken for 5 cycles of 1 min each, with a 30 s cooling break between cycles. The resulting suspension was transferred to a 1.5 mL sterile Eppendorf tube, and the procedure continued according to the kit’s protocol. The obtained RNA was resuspended in 100 µL of ultrapure water (A&A Biotechnology, Gdansk, Poland).

### 2.3. Protocol No. 2

The RNA isolation was conducted according to the Total RNA Mini Plus protocol (A&A Biotechnology, Gdansk, Poland). The modification was made only in the sample preparation stage rather than grinding the mycelium in a mortar with liquid nitrogen as recommended by the manufacturer; the mycelium was vortexed for 2 min at 4000 rpm in a tube containing zirconium beads with the addition of 400 µL of Fenozol Plus (A&A Biotechnology, Gdansk, Poland). The obtained RNA was resuspended in 100 µL of ultrapure water. While grinding the mycelium in a mortar is an effective method for its disruption, it is inefficient and impractical when working with a larger number of samples.

### 2.4. RNA Clean-Up

The isolated RNA was subjected to a purification process following the Clean-Up RNA Concentrator protocol (A&A Biotechnology, Gdansk, Poland). The purified RNA was resuspended in 20 µL of ultrapure water (A&A Biotechnology, Gdansk, Poland). The quantity and purity of the obtained RNA after purification were assessed using a NanoDrop spectrophotometer (Thermo Fisher Scientific) by measuring absorbance at a wavelength of 260 nm and calculating the 260 nm/280 nm and 260 nm/230 nm ratios. Table 1 presents the concentration measurements of isolated RNA before and after the purification procedure. The average mass of the sample used for RNA isolation is indicated in Table 2 and Table 3.

## 3. Results

### 3.1. RNA Integrity

**Figure 1:**
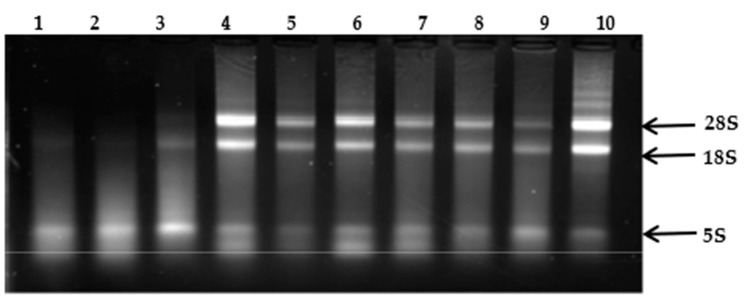
RNA isolated from *Penicillium crustosum* (1–6); *Penicillium rubens* (7–10).

**Figure 2:**
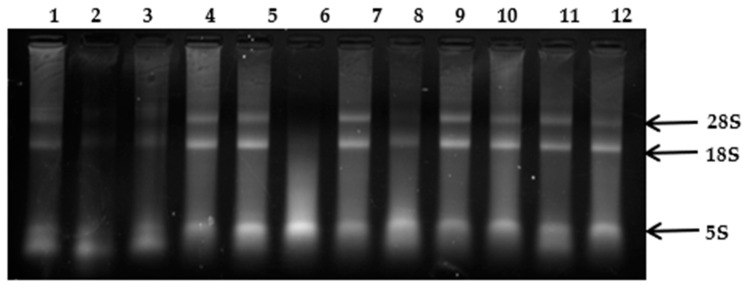
RNA isolated from Penicillium griseofulvum (1–6); Aspergillus fumigatus (7–10).

The visualization of the electrophoretic separation of purified RNA (Figure 1 and Figure 2) allows for a preliminary assessment of RNA degradation. Based on the electrophoretic separation, it was determined that the quality of RNA isolated according to Protocol 1 is superior to that isolated using Protocol 2. The electrophoretic separation of RNA isolated with Protocol 1 shows distinct bands corresponding to 28S, 18S, and 5.8S rRNA, indicating better RNA integrity. Figure 1 and Figure 2 present the electrophoretic separation results of the isolated RNA from various species and strains, each in triplicate. In lanes 1 to 6 of Figure 1, the RNA isolated from two different strains of *P. crustosum* is shown, while lanes 7 to 10 depict the electrophoretic separation of RNA isolated from two strains of *P. rubens*. In Figure 2, lanes 1 to 6 present the electrophoretic separation of RNA isolated from *P. griseofulvum*, and lanes 7 to 12—the separation of RNA isolated from *A. fumigatus*.

### 3.2. RNA Concentration

The RNA obtained using the isolation method from Protocol 1 is suitable for use as a substrate in cDNA synthesis reactions. In contrast, the concentrations of purified RNA isolated according to Protocol 2 are insufficient for cDNA synthesis.

Referring the results to the average concentration of RNA after purification in relation to the dry mass of the material from which it was isolated, the results obtained by means of Protocol no 1 are presented in Table 2 and Protocol no 2 in Table 3.

### 3.3. RNA Purity

RNA is considered pure if the A260/A280 and A260/A230 ratios are greater than 1.8. If the A260/A280 ratio is below this threshold, it indicates that the sample is contaminated with proteins. In contrast, an A260/A230 ratio below 1.8 suggests the presence of residual organic compounds, such as phenol [10].

Due to the insufficient purity of RNA isolated according to Protocol 2, as the A260/A230 ratios for each sample were below 1.8, and the RNA concentration was too low for further analysis, these samples were disqualified from further use. Table 4 presents the A260/A280 and A260/A230 values for RNA isolated according to Protocol 1.

## 4. Discussion

The effectiveness of ribonucleic acid analysis depends on RNA quality, and thus on the selection of an isolation method with the best qualitative and quantitative characteristics. In the case of filamentous fungi, RNA isolation is particularly challenging due to their structure, and especially the time-consuming process of cell wall disintegration. For this reason, isolation protocols are developed or selected to be most optimal for specific species of filamentous fungi [11,12,13].

To select the most optimal RNA isolation method for fungi from the genera *Penicillium* and *Aspergillus*, two isolation methods were tested. Both protocols were based on the action of Fenozol Plus, a mixture of phenol and chaotropic salts, which leads to cell lysis and the inactivation of native RNases. The key step for the efficiency of RNA isolation is the disintegration of the cell wall in filamentous fungi. It is important to emphasize that mechanical methods are preferred, as enzymatic lysis, due to the specific conditions required, carries the risk of RNA degradation, as such a condition can also be optimal for RNase activity [13].

In the tested protocols, two different mechanical methods were applied for the lysis of the cell wall in selected species of *Penicillium* and *Aspergillus* and the same clean-up protocol. It should be emphasized that the amount of RNA required for reverse transcription typically falls within the range of 0.1–5 μg, and the final volume of the reverse transcription reaction is limited. This limitation depends on the reagents used and establishes a threshold RNA concentration necessary for a sample to be suitable for this reaction. The results clearly indicate that higher yields can be obtained using the bead-beater method compared to manually vortexing the sample. The higher efficiency observed with the bead-beater method (Protocol 1) is achieved even though the such vigorous shaking generates high temperatures, which can promote RNA degradation. Therefore, it is crucial to perform the shaking in the presence of Fenzol Plus, which deactivates RNases present in the biomass, and to introduce cooling breaks during the shaking process. The efficiency of the method (Protocol 2) using manual vortexing in terms of the amount of RNA obtained was significantly lower—approximately 2.2 times lower before purification. It appears that the main reason for this is the structure of the filamentous fungal cell wall and the difficulty in mechanically lysing it. In this case, the vortexing method leads to the lysis of the cell wall in only a portion of the cells in the culture.

Another important step affecting the efficiency of the RNA isolation process is the separation of RNA from DNA, proteins, and other organic contaminants. In Protocol 1, RNA extraction was carried out using chloroform, while in Protocol 2, ultrapure water was used. Both water and chloroform aim to separate the mixture into fractions, effectively isolating DNA, proteins, polysaccharides, and polyphenols from RNA. The results indicate that using ultrapure water (Protocol 2) successfully separates DNA and proteins from RNA; however, the A260/A230 absorbance values suggest that the sample remains significantly contaminated with polysaccharides and polyphenols. The use of chloroform (Protocol no. 1) causes smaller DNA fragments, proteins, and polysaccharides to settle in the lower fraction during centrifugation, where they undergo partial dissolution. Longer DNA fragments accumulate at the phase interface, while RNA remains in the upper aqueous fraction, which can be easily collected and subsequently precipitated by the addition of isopropanol. The effectiveness of using chloroform, in terms of the quality of the obtained RNA, is confirmed by absorbance results, with both A260/A280 and A260/A230 ratios exceeding 2 and 1.8, respectively. The efficacy of the RNA isolation combined with purification ranges from 22 to 96 µg/g of dry mass of mycelium. However, it should be emphasized that the NanoDrop spectrophotometer measures total nucleic acids, so any contaminating DNA in the sample can lead to an overestimation of the RNA concentration. This is especially problematic when RNA samples are not treated with DNase during the isolation process. On the other hand, the presence of contaminants such as phenol, chaotropic salts, or reagent residues can also affect absorbance values. Therefore, RNA concentration results should be interpreted with these limitations in mind. To confirm the RNA integrity and purity, we used agarose gel electrophoresis, where the presence of degradation products or DNA contamination can be visually assessed.

In conclusion, considering the structure of fungi from the *Aspergillus fumigatus* and *Penicillium genus*, the most efficient method that also yielded a product of high purity and integrity was the one combining mechanical lysis with a bead-beater and RNA extraction with chloroform. Due to the use of a bead-beater and relatively small volumes of chloroform, the protocol can be applied in routine laboratories with a high specimen load.

## Figures and Tables

**Table 1:** RNA concentration.

Species	Method											
	Protocol No 1 [ng/µL]						Protocol No 2 [ng/µL]					
	BC	AC	BC	AC	BC	AC	BC	AC	BC	AC	BC	AC
***P. crustosum***	60.6	**184.7**	74.4	**143.5**	63.9	**202.6**	46.3	**33**	62.0	**57.6**	60.9	**28.0**
***P. crustosum***	223.7	**831.4**	195.3	**385.2**	190.4	**491.6**	81.9	**95.1**	52.5	**37.3**	76.7	**82.7**
***P. rubens***	153	**319.5**	110.6	**38.1**	148.3	**523.8**	60.2	**31.1**	68.3	**30.6**	50.3	**82.7**
***P. rubens***	154.9	**334.6**	92.9	**40.8**	265.4	**1260**	41	**26.4**	49.3	**11.1**	65.3	**75.5**
***P. griseofulvum***	204.8	**105.8**	270.4	**361.9**	192.5	**98.5**	51.3	**42.7**	60.3	**63.9**	76.7	**57.0**
***P. griseofulvum***	151	**175**	47.8	**90.4**	58.9	**148**	50.1	**41.1**	41.9	**58.4**	59.9	**98.5**
***A. fumigatus***	262.1	**741.2**	68.6	**67.1**	252.3	**741.2**	129	**153**	114	**121**	143	**99.1**
***A. fumigatus***	87.1	**238.2**	100.1	**239.2**	111.9	**286.5**	95.7	**23.6**	65.5	**43.7**	59.9	**104**

(BC—before clean up; AC—after clean up).

**Table 2:** The average yield of RNA isolation obtained by means of Protocol no 1.

Species	Concentration [ng/µL]	Mass [g]	RNA/Dry Mass [µg/g]
***P. crustosum***	176.93	0.117	~30
***P. crustosum***	569.4	0.118	~96
***P. rubens***	293.8	0.195	~30
***P. rubens***	545.13	0.196	~55
***P. griseofulvum***	188.73	0.113	~33
***P. griseofulvum***	137.8	0.122	~22
***A. fumigatus***	516.5	0.195	~52
***A. fumigatus***	254.63	0.108	~47

**Table 3:** The average yield of RNA isolation according to Protocol no 2.

Species	Concentration [ng/µL]	Mass [g]	RNA/Dry Mass [µg/g]
***P. crustosum***	39.5	0.117	~7
***P. crustosum***	71.7	0.118	~12
***P. rubens***	48.1	0.195	~5
***P. rubens***	36.7	0.196	~4
***P. griseofulvum***	57.5	0.113	~10
***P. griseofulvum***	66.00	0.122	~11
***A. fumigatus***	124.4	0.195	~13
***A. fumigatus***	57.1	0.108	~11

**Table 4:** Purity of RNA isolated according to Protocol 1.

Species	Ratio					
	A_260_/A_280_			A_260_/A_230_		
***P. crustosum***	2.19	2.22	2.23	2.12	2.11	1.74
***P. crustosum***	2.21	2.14	2.18	2.05	1.69	2.10
***P. rubens***	2.20	2.20	2.20	1.93	0.83	1.77
***P. rubens***	2.17	2.12	2.22	1.79	0.82	2.27
***P. griseofulvum***	2.14	2.11	2.10	0.75	1.99	0.77
***P. griseofulvum***	2.10	2.06	2.20	1.58	1.65	1.98
***A. fumigatus***	2.24	2.20	2.17	2.17	0.55	2.04
***A. fumigatus***	2.21	2.19	2.16	2.17	2.01	1.59

## Data Availability

The original contributions presented in this study are included in the article. Further inquiries can be directed to the corresponding author.
